# Using an innovative family-centered evidence toolkit to improve the livelihood of people with disabilities in Bamenda (Cameroon): a mixed-method study

**DOI:** 10.3389/fpubh.2023.1190722

**Published:** 2024-01-31

**Authors:** Mirabel Nain Yuh, Gloria Akah Ndum Okwen, Rigobert Hanny Pambe Miong, Nicola Luigi Bragazzi, Jude Dzevela Kong, Zahra Movahedi Nia, Tetamiyaka Tezok Kinlabel, Okwen Patrick Mbah

**Affiliations:** ^1^Effective Basic Services (eBASE) Africa, Bamenda, Cameroon; ^2^Africa Evidence Network (AEN), Johannesburg, South Africa; ^3^The Partnerships for Inclusive Research and Learning (PIRL) Project, Toronto, ON, Canada; ^4^Department of Mathematics and Statistics, Faculty of Science, York University, Toronto, ON, Canada; ^5^Africa-Canada Artificial Intelligence and Data Innovation Consortium, Toronto, ON, Canada; ^6^Department of Information Technology, University of Ghana, Accra, Ghana; ^7^Africa Centre for Evidence (ACE), University of Johannesburg, Johannesburg, South Africa

**Keywords:** disability, financial instability, inclusive policies, improving livelihood, evidence portals, evidence ecosystem, income-generating activities, innovation and best practices

## Abstract

**Background:**

Most of the disability-related scholarly literature focuses on high-income countries, whereas there is a lack of data concerning challenges (barriers and obstacles) and opportunities (participatory research and community engagement) in the Global South. Moreover, many frameworks for interventions for people with disabilities (PWDs) have been designed for resource-rich contexts, and little is known about their translatability to low- and middle-income countries (LMICs).

**Objective:**

The main objective of this study was to design and pilot an interventional approach based on an innovative framework aimed at improving the livelihood of PWDs in LMICs.

**Methodology:**

The present mixed-method study was conducted in Bamenda, North-West Region of Cameroon, through an intervention of household visits by community health workers using innovation and best practices informed by a systematic literature review and embedded into an evidence toolkit called the eBASE Family-Centered Evidence Toolkit for Disabilities (EFCETD), adapted from the WHO matrix and consisting of 43 questions across five categories (health, education, social wellbeing, empowerment, and livelihood). Out of 56 PWDs identified, 30 were randomly sampled, with an attrition of four participants. Three datasets (baseline, qualitative, and quantitative) were collected. The Washington Group tool, exploring the type of disability, gender, how long one has had the disability, their facility situation coupled with their coping strategies, and the context of livelihood, was used to design the questionnaire for baseline data collection. Qualitative data were collected through key informant interviews and focus group discussions analyzed with MAXQDA, while quantitative data were collected through the EFCETD and analyzed by means of descriptive statistics.

**Results:**

In total, 69.2% of PWDs were female individuals. Many PWDs were aged 10–20 years (57% of the sample size). Physical/motor disability was the most common type of disability recorded (84.6%). The mean percentile for education increased from 29.9% during the first visit to 70.2% during the last visit, while the mean percentile for health increased from 65.4 to 78.7% and the mean percentile for social wellbeing moved from 73.1 to 84.9%. The livelihood and empowerment standards increased from 16.3 to 37.2% and from 27.7 to 65.8%, respectively. Overall, the temporal trend was statistically significant (*F* = 35.11, *p* < 0.0001). The adjusted score increased from the baseline value of 45.02 ± 2.38 to 61.07 ± 2.25, 65.24 ± 2.67, and 68.46 ± 2.78, at 4, 8, and 12 months, respectively. Compared to the baseline, all timepoints were significantly different, indicating a significant impact of the intervention, which became stable after 4 months and was preserved until 12 months.

**Conclusion:**

PWDs faced many endeavors for sustainability and challenges resulting from a lack of inclusive policies and practices, leading to their exclusion from education, employment, and healthcare. Using implementation science approaches could bridge the gap and make policies and practices more effective.

## Introduction

Most of the disability-related scholarly literature focuses on high-income countries, even though, according to the “World Health Organization” (WHO), approximately 80% of the 1 billion people living with various forms of disabilities worldwide are based in low- and middle-income countries (LMICs) (1–3). A large portion of them (between 93 and 150 million) (3) are children, who are more likely to drop out and not complete primary education, compared with their able-bodied counterparts (4, 5). Despite the relevance of this topic, there is a lack of data concerning challenges (in terms of structural and perceived barriers and obstacles) and opportunities (in terms of participatory research and community engagement), when facing disability in the Global South. Furthermore, even though scholarly interest in disability as lived and experienced in LMICs has been increasing in the last years, most of the available research utilizes concepts, theories, and practices borrowed from studies conducted in the Global North, thus potentially increasing the epistemological and methodological vulnerabilities of disability-related investigations carried out in the global periphery (6).

In LMICs, families and, in particular, women are most responsible for the care of persons with disabilities (PWDs) (7, 8). Both PWDs and their families are still stigmatized and subjected to high levels of exclusion in most areas, especially in rural and underserved settings. PWDs basically experience economic and financial hardship, poor access to basic healthcare and educational services, and implementation bottlenecks (9). PWDs face many endeavors for sustainability and challenges resulting from a lack of inclusive policies, leading them to further marginalization and exclusion from social and economic activities, such as education, employment opportunities, healthcare, and sustainable livelihoods (10–13).

Cameroon is one of the LMICs in which disability poses considerable challenges (14). Studies conducted by Cockburn et al. (15) and Foti et al. (16), reviewed by Ray et al. (17) and by Cannata et al. (14), revealed that the population-based prevalence rate of PWDs in the North-West Region of Cameroon and in the entire country was approximately 6.2% and 6.1–12.9%, respectively, with PWDs being often left behind the evidence-based practice training (18). The disability prevalence rate was strongly associated with age, and some disabilities were more prevalent in specific age groups: for instance, physical and hearing impairments were the most commonly reported health conditions in children, while physical impairments, epilepsy, and hearing impairments were the most common among adults, aged 18–49 years. In adults aged 50 years and older, the most prevalent impairments were hearing, vision, and physical impairment, followed by depression and epilepsy (15, 19).

Available evidence shows that supporting families to cope with children with disabilities through innovation and best practices is a stepwise approach to inclusive development and equity in participatory implementation strategies aimed at improving livelihoods in families with PWDs (9). Community-based rehabilitation strategies endorsed by the WHO and other international bodies suggest enhancing the quality of life of these persons and their caregivers by trying to meet their basic needs and ensuring inclusion and participation using predominantly local resources (20, 21). These interventions incorporate five broad components, i.e., health, education, livelihood, social aspects, and empowerment (22).

However, as previously mentioned, even though developing interventions aimed at economically empowering children and adults with impairments and disabilities for livelihood sustainability is expected to be of paramount importance, most of the frameworks for interventions on PWDs have been designed and implemented in resource-rich contexts, and little is known about their translatability to LMICs. When inclusive development-related perspectives have been applied in LMICs, they have been substantially drawn from the first wave of the evidence revolution as part of the so-called “New Public Management” course, categorized as “results agenda” (or “outcome monitoring”), which, according to White (23), characterized the nineties and in high-income countries were followed by further waves called “rise of impact evaluation,” “rise of systematic reviews,” and “knowledge brokering.”

Diversified economic empowering needs of PWDs in LMICs, including Cameroon, have been, however, overlooked and can be still largely ascribed to the societal norm of ableism according to Lorenzo et al. (24, 25), who demonstrated how community health workers empowered and trained to be(come) community disability workers can highly contribute to social justice for PWDs and their families in accordance with evidence-based experiences conducted in three southern African countries. Based on recent literature reviews, replicating this evidence in other LMICs, and, in particular, in Cameroon, with the use of community health workers to catalyze the process of improving the livelihood of PWDs would be very impactful and is anticipated to provide better outcomes in these countries.

The present article attempts to fill in these knowledge gaps, by designing and piloting an interventional approach based on an innovative framework aimed at improving the livelihoods of PWDs in Bamenda, North-West Region of Cameroon. Moreover, in detail, we used innovation and best practices embedded into an evidence toolkit called the eBASE Family-Centered Evidence Toolkit for Disabilities (EFCETD) in order to enhance the quality of life for children and adults with disabilities in a region of Cameroon.

## Methodology

### Study area

This study was conducted in the city of Bamenda, North-West Region of Cameroon. Bamenda is the third largest city in Cameroon with approximately 573,000 inhabitants (approximately 2.6% of Cameroon’s population). Bamenda is geographically located between latitude 5.963051 and longitude 10.159121 with GPS coordinates of 5° 57′ 46.9836″ N and 10° 9′ 32.8356″ E. The elevation of Bamenda is 1282.285.^1^ Specifically, data were collected within 12 neighborhoods in Bamenda: Mile 03 Nkwen, Mile 04 Nkwen, Ntankar, Chindeh, Alahkuma, Mile 06 Mankon, Mbengwi Road, Buea Bamenda Street, Foncha Street, Ntahmuche, Nimbung, and Old town.

According to Cameroon’s National Observatory of Public Health, Ministry of Public Health, as of 2015, Cameroon had a population of 22,179,707 individuals, with 43% aged 0–14 years, 53.5% aged 14–64 years, and 3.5% aged 65 years and older. From a demographic standpoint, as the age pyramid exhibits a rather broad base and a narrow or pointed top with a triangular shape, the country shows characteristics typical of developing countries, which are mostly comprised of young populations (in Cameroon, approximately 62.5% of the entire population), with high natality and high mortality rates. Bamenda reports similar trends as well in terms of age groups.

### Study population

Reflecting the previously described age pyramid, our study population is comprised of children and adults living with disabilities in the age group of 2–38 years.

### Ethical approval

The study project protocol received full ethical clearance (approval code eBASE_IRB/02/2019). Informed written consent was sought from study participants and/or their parents or legal guardians.

### Study design and sampling technique

This study leveraged a mixed-method approach conducted using a series of structured questionnaires, and its administration was undertaken in Bamenda, Cameroon, with an initial sample of 56 PWDs. A snowballing sampling was applied to recruit and interview eligible participants. This technique was based on identifying a disabled person through a stakeholder meeting, self-identification, referral by a service provider community member, or referral by families with PWDs.

To ensure the representativeness and generalizability of our findings, 30 PWDs were randomly sampled from the initial sample. The attrition of four participants was recorded during the follow-up visits. As such, the final sample consisted of 26 PWDs.

### Inclusion criteria

Inclusion criteria for being eligible to take part in the study were reporting any form of impairment (physical/motor, sensory, mental, intellectual, developmental, or relational). The definition of disability was based on the “Washington Group on Disability” (WGD) and the United Nations (UN) “Convention on the Rights of Persons with Disabilities” (UNCRPD; accessible at https://www.ohchr.org/en/instruments-mechanisms/instruments/convention-rights-persons-disabilities#:~:text=Persons%20with%20disabilities%20include%20those,an%20equal%20basis%20with%20others), according to which PWDs are people who have “long-term physical, mental, intellectual, or sensory impairments which in interaction with various barriers may hinder their full and effective participation in society on an equal basis with others.”

### Data collection

Three datasets of data were collected, namely, (i) baseline, (ii) qualitative, and (iii) quantitative data, using dedicated software, including MAXAPP and MAXQDA software, between April 2019 and January 2021. A stakeholder engagement session meeting with the Ministry of Health, social groups for people with special needs, social services, council members, and major civil society organizations (CSOs) was organized to brainstorm, develop research questions, design disability-related projects, and identify and recruit PWDs. Furthermore, a rapid systematic review of the literature was conducted to provide methodological guidance for the development of the disability study. Moreover, in detail, a rapid review of best practices was done to identify interventions aimed at effectively improving livelihoods for PWDs. The PICO framework was devised to inform the literature screening, with the population of interest (P) consisting of children and adults with disabilities, undergoing interventional programs (I) based on best practices aimed at improving their livelihoods at the household and community level, compared against current practices (C). Outcomes of interest (O) were improvements in livelihoods as measured by needs met and wellbeing enhanced. Several evidence-based practice databases were searched, including the Campbell Collaboration, the Cochrane Library, the Joanna Briggs Institute (JBI), and the “International Initiative for Impact Evaluation” (3ie) Evidence Hub. Moreover, in addition to these global resources and repositories, a locally relevant and informed database was searched, namely the “Africa Evidence Network” (AEN).

In addition, searching was also carried out on governmental databases and development agencies alongside sending emails to relevant authors and experts in the field. We synthesized available evidence, and out of 2,124 studies conducted between 2007 and 2018, 153 were eligible for our purpose. These consisted of one national policy document, one guideline, 53 systematic reviews, and 95 primary studies. Policy documents included the national policy for the rights of PWD, the WHO policies of Community-Based Rehabilitation, the UNCRPD, and the UN “Sustainable Development Goals” (SDGs).

### Baseline data collection

The Washington Group tool was used to design the questionnaire for baseline data collection. The questionnaire addressed several variables related to PWD, such as their age, sex/gender, type of disability, how long they have had the disability, and their physical/mental conditions coupled with their coping strategies and functional performance. Moreover, this questionnaire explored the context of livelihood for children and adults living with disabilities.

After gathering the experiences and current practices, a systematic search of evidence on best practices to improve the livelihoods of PWDs at the household and community level was carried out.

### Development of the family-centered evidence toolkit for disabilities

A toolkit was developed by linking the current practices to best practices on a Microsoft Word document, combining sustainability (i.e., SDGs), evidence, and best practice recommendations.

The toolkit was built through MAGPI, where five different categories were created with 43 questions. These categories were adapted from the WHO matrix, including health, education, social wellbeing, empowerment, and livelihood (20). Five community healthcare workers were recruited through the Bamenda health area. They were equally trained on how to use the toolkit, email system, and WhatsApp and how to collect data for the project. The toolkit was piloted with community health workers, and, in turn, the community health worker further piloted the toolkit in their neighborhood. Research team members and community health workers, then, reconveyed in another session to identify doubts and criticisms, and checklists were prepared to standardize the procedure.

### Qualitative data collection

Qualitative data were collected through key informant interviews (KIIs) and focus group discussions (FGDs) guided by a questionnaire tool. The recording was done through MAXAPP and WhatsApp audios and later sent to the eBASE team.

### Quantitative data collection

Quantitative data were collected through household visits every 2/3 months by community health workers, spanning a period of 12 months. EFCETD was installed on the smartphones of community health workers for data collection. Contacts and locations of households of children with disabilities were divided among the community health workers such that each community health worker was responsible for visiting at least seven households twice every 2 or 3 months. Appointments were scheduled with the households by the community health workers to administer the toolkit and carry out a follow-up procedure.

Participants were interviewed with questions guided by the toolkit and recorded. Community health workers also educated the families on how to improve the livelihood of PWDs by providing counseling on evidence-based recommendations on how to manage and care for their children and adults with disabilities. After four household visits, each family was assessed based on the toolkit questions and scored. Then, the scores on the toolkit were downloaded into a spreadsheet for further processing and analysis. Since not all questions of the toolkit were applicable to every PWD recruited in the study, both raw and adjusted scores for each domain were computed. Moreover, in detail, an adjusted score was calculated as the total score adjusted per the maximum score achievable, taking into account the covariates (age and type of impairment). From these domains, an overall score was finally derived. Each family that scored above the cut-off score of 60 points was rewarded with seed funding as an incentive for an income-generating activity for livelihood.

### Computer-assisted data management

Qualitative data collection was performed using MAXAPP on Tecno smartphones. Transcription, coding, and data analysis were conducted using MAXQDA Analytics Pro version 2022.

### Qualitative data management using computer-assisted technology (MAXAPP and MAXQDA)

Qualitative data were recorded through MAXAPP, and the audios were sent to the eBASE server to be downloaded at the head office by authorized eBASE staff members. The audio files were uploaded, transcribed, and saved on Dropbox. The transcripts were then coded using MAXQDA Analytics Pro version 2022. Several codes and subcategories were developed. The codes included barriers, facilitators, coping strategies, health, education, social wellbeing, livelihood, and empowerment. In addition, there were codes on conflict and disability, sexual reproductive health rights (SRHRs), and current and best practices.

### Quantitative data analysis using computer-assisted technology

Quantitative data were collected by community health workers during household visits through the MAGPI application installed on their smartphones guided by the toolkit (EFCETD) used to score the families based on their responses to a series of questions on engagement and compliance with evidence-based recommendations in the care of their children living with disabilities. A database was generated using Microsoft Word Excel 2013, and a descriptive statistical analysis was performed. In addition, a statistical analysis was conducted to assess the impact of the intervention and quantify the temporal trend of the various follow-up visits performed, using STATA version 16.0.

## Results

### Study population

From a quantitative standpoint, the main demographic characteristics of the study population (n = 26) are reported in Table 1. Eighteen PWDs were female individuals (69.2% of the entire population), while eight were male individuals (30.8%). In terms of distribution of age groups, seven (26.9%) were aged between 0 and 9 years, fifteen (57.7%) between 10 and 20 years, two (7.7%) between 21 and 30 years, and 2 (7.7%) between 31 and 40 years. Twenty-two PWDs (84.6%) reported a physical or motor impairment, while seven (26.9%) and three (11.5%) reported mental disability and a sensory (hearing) impairment, respectively. No cases of visual impairment as well as of intellectual, relational, or developmental disability were recruited.

**Table 1:** Demographic characteristics of participants in the study (*N* = 26).

Variable	Level	Number	Percentage
Sex/gender	Female	18	69.2%
	Male	8	30.8%
Age groups	0–9	7	26.9%
	10–20	15	57.7%
	21–30	2	7.7%
	31–40	2	7.7%
Type of disability/impairment	Physical or motor impairment	22	84.6%
	Sensory (visual) impairment	0	0.0%
	Sensory (hearing) impairment	3	11.5%
	Mental disability	7	26.9%
	Intellectual disability	0	0.0%
	Developmental disability	0	0.0%
	Relational disability	0	0.0%

As previously mentioned, the community healthcare workers visited the families four times over a period of 12 months with an interval of 2 or 3 months. Attrition was recorded during the four rounds of follow-up visits, with four participants lost with respect to the baseline visit. The main reason for the attrition was the constant displacement of participants during the time of visits due to the socio-political crisis that was afflicting the study area.

### Qualitative results

Among the various themes identified, one of the most relevant was about the stories experienced by families and PWDs, and the barriers influencing their livelihood and wellbeing, including obstacles to meeting educational needs. According to the WHO guidelines, education is, indeed, one of the components that most influence the quality of life of PWDs in both the short and long term. Study participants mentioned that, due to political instability and insecurity, governmental institutions have shifted their priorities, addressing immediate security concerns over investing in high-quality education, leading to changes in educational policies and regulations and resulting in a lack of resources for inclusive educational programs for children with disabilities. Inclusive education requires dedicated funding and long-term planning: the provision of special education services is, instead, disrupted and underfunded, with several schools lacking the necessary infrastructure, trained teachers, and specialized materials to provide inclusive education. Moreover, families with children with disabilities may face higher medical and caregiving costs, which can limit their ability to afford school-related expenses, such as uniforms, transportation, and assistive devices. Poverty can be a significant barrier to accessing education, more specifically inclusive education. The combination of all these barriers can create a cycle of exclusion for children with disabilities, magnifying disparities and inequities. In conclusion, political crisis (62.5%), insecurity (50.0%), and financial issues (50.0%) were the three most frequently reported barriers for children with disabilities to achieving adequate educational levels, followed by transportation (37.5%), lack of dedicated facilities (12.5%), medical support (12.5%), and stigma (12.5%). Unmet educational needs are a lack of adequate preparation for school (40.0%), dearth of infrastructure (20.0%), specialized teachers (20.0%), high fees (20.0%), and medical needs (20.0%), as indicated by the following excerpts of the conversations with the study participants.

*“She has been affected by the crisis in that, if there were no crisis she would have been going to school and this would have helped her improve in some aspects of her physical skills.”*

*Mother of a child with a disability (KII, Bamenda, Cameroon).*

*“I really wish/ like him to go to school but with the crisis, he cannot, because of transportation problems it is not possible to go and come. Then, also considering the means for us to work, get the money, and send him to school, well, all this is not available/possible.”*

*Mother of a child with a disability (KII, Bamenda, Cameroon).*

*“It is very challenging for me because it is difficult to transport her to and from school…”*

*Mother of a child with a disability (KII, Bamenda, Cameroon).*

*“Yes, I wanted to say that it is not easy because they (i.e., children with disabilities) have their own needs. They have a special way of learning, which requires special methods of teaching that are not delivered in normal schools. Some of them do not talk, and with some of them, you need to use sign language. So, you cannot take them to a normal school. There can be a school around you, but it is difficult to take your child there because they will not be able to cope with your child.”*

*Mother of a child with a disability (FGD, Bamenda, Cameroon).*

*“If our kids have a physical handicap, it may be challenging for them to access normal schools, given how they have been built. There are some classes you must take the stairs to reach them. If a child uses a wheelchair, it will not be possible for them to climb the stairs. You may have to beg someone to help you. You may even beg a teacher, but sometimes they are not willing to help you. So, normal schools do not have the facilities to accommodate children with disabilities.”*

*Mother of a child with a disability (FGD, Bamenda, Cameroon).*

*“…Also, some of our children who are mentally challenged, when they go to the normal school, become like a source of attraction for others, who want to look at them and mock them all the time. This makes the persons with disabilities get sad and angry. I am saying this because I have taken my daughter several times to school, but she wasn’t happy at all … however, as soon as we walk into her special needs school, she is always excited and happy.”*

*Mother of a child with a disability (FGD, Bamenda, Cameroon).*

According to the WHO, medical needs are a fundamental part of the components influencing the wellbeing and livelihood of PWDs. This is confirmed by the stories of the participants, which reveal that most PWDs are in need of physiotherapy (75.0%), medications (50.0%), wheelchairs (50.0%), special diets (25.0%), walkers (25.0%), and crutches (25.0%), to improve their wellbeing. These needs can vary depending on the type and severity of the disability, but they are essential for enhancing the overall quality of life, in terms of improved health conditions, autonomy, and social inclusion, for many PWDs. Some relevant quotes by participants were:

*“It is quite difficult to pay for her medical bills, but her father has been trying, making a lot of efforts. However, it was so difficult that, at a certain time, we could not provide high-quality medical support to her anymore.”*

*Mother of a child with a disability (KII, Bamenda, Cameroon).*

*“I think if she could have a wheelchair, this would help a lot.”*

*Mother of a child with a disability (KII, Bamenda, Cameroon).*

*“I used to think that government can or is supposed to cover hospital expenses like, for example, if you go to a government hospital, but that was not the case when my daughter had an accident. She fell and broke her hand. I went to the hospital with her disability card, and I was expecting that they would do the X-ray and all other examinations without me having to pay a dime. But, since the procedure would have been extremely lengthy and we had to wait in the queue, looking at the child crying in my hands, I just decided to pay. Even if the government wants to do something, they put bottlenecks everywhere.”*

*Mother of a child with a disability (FGD, Bamenda, Cameroon).*

### Quantitative results

**Figure 1:**
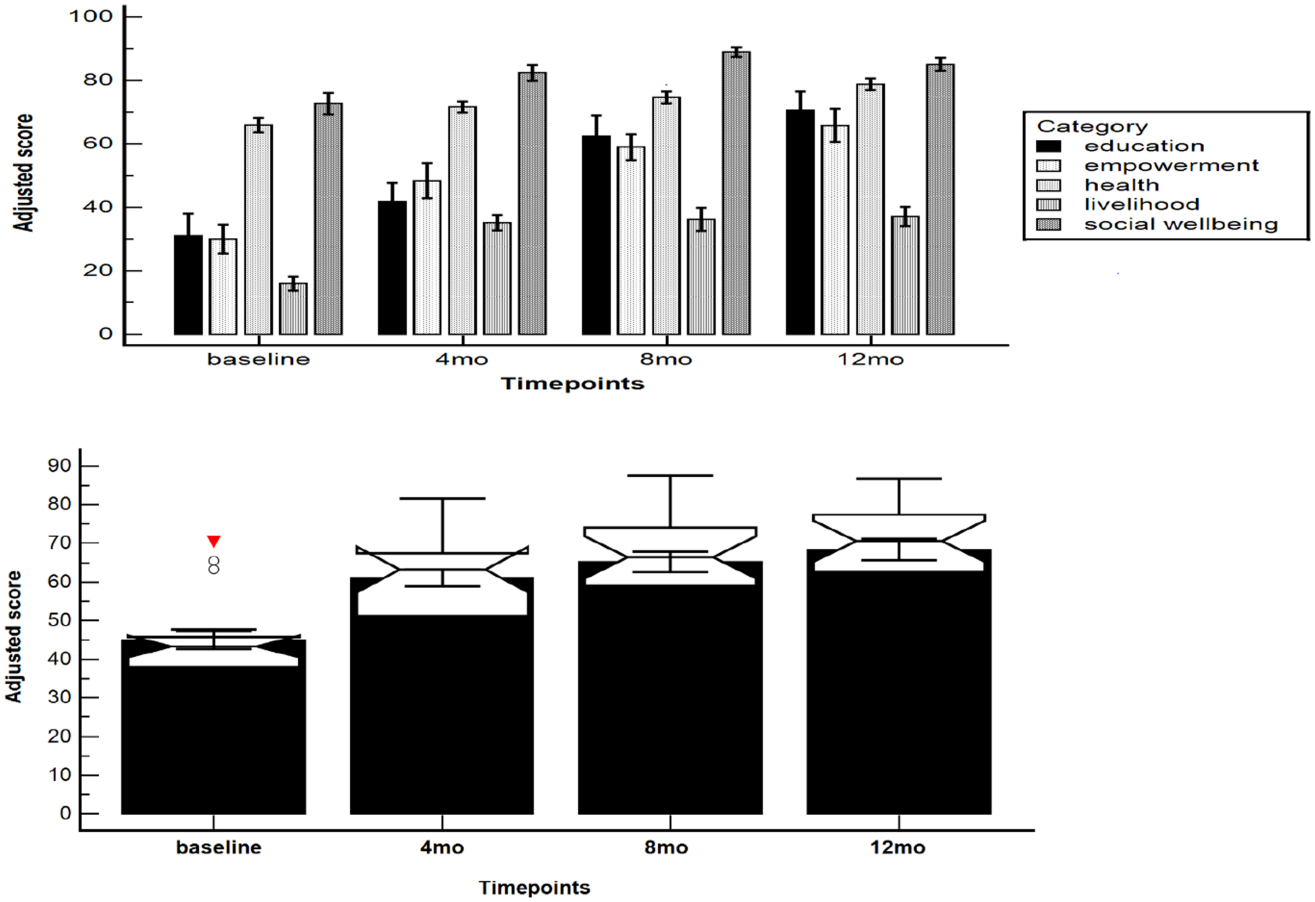
Adjusted mean (top) value scores of participants during household visits in Bamenda (Cameroon) broken down according to each category/domain (education, health, social wellbeing, livelihood, and empowerment), and overall (bottom).

Figure 1, top, pictorially represents the adjusted means of education, health, social wellbeing, livelihoods, and empowerment during each household visit, while adjusted overall scores are represented in Figure 1, bottom.

Overall, the temporal trend was statistically significant (*F* = 35.11, *p* < 0.0001). The adjusted score increased from the baseline value of 45.02 ± 2.38 [95%CI 40.01–50.02] to 61.07 ± 2.25 [95%CI 56.35 to 65.79], 65.24 ± 2.67 [59.63 to 70.85] and 68.46 ± 2.78 [95%CI 62.63 to 74.29], at 4, 8, and 12 months, respectively. Compared with the baseline, all timepoints were significantly different: the mean difference was computed at −16.05 ± 2.59 ([95%CI −23.72 to −8.38], *p* < 0.0001) at 4 months, −20.22 ± 2.86 ([95%CI −28.69 to −11.75], *p* < 0.0001) at 8 months, and −23.44 ± 2.88 ([95%CI −31.97 to −14.92], *p* < 0.0001) at 12 months. This indicated a statistically significant impact of the intervention, which became stable after 4 months and was preserved until 12 months.

## Discussion

PWDs represent one of the largest minority groups in the world, estimated to be over 15% of the world’s population, often left out in development processes and progress (26). A substantial portion of the global disability population comprises children, residing in LMICs. However, there exist significant gaps in research data related to disability in the Global South, in terms of the challenges faced by PWDs in these regions, including both structural and perceived barriers and obstacles. This data gap warrants more comprehensive research efforts, to tackle north–south disparities, by capturing local unique experiences and realities, instead of over-relying on concepts, theories, and practices borrowed from studies conducted in the Global North. To overcome the epistemological and methodological vulnerabilities that afflict most of the disability-related investigations carried out in LMICs, the present study was undertaken to provide data on the current wellbeing and challenges faced by children and adults with disabilities in the Global South (Bamenda, Cameroon). Our qualitative findings suggest that PWDs are still stigmatized and marginalized, facing exclusion and experiencing disparities. These findings are well in line with some studies conducted by the United Nations, which show that great misconceptions still exist about PWDs (10). These misconceptions hinder the design and implementation of inclusive development programs. Counseling and early sensitization of families including parents and service providers in the dissemination and translation of knowledge and rehabilitative care are anticipated to set the trend for a positive change based on the best available evidence.

Through the intervention of household visits by community health workers, this study has aimed to address this gap by providing counseling and guidance in order to improve the livelihoods of PWDs using innovation and best practices embedded into an evidence toolkit that we call EFCETD. These practices were adopted in conformity with past interventions administered by the WHO (20) aimed at enhancing the quality of life of PWD. According to White (23), a specific intervention cannot be assumed to work effectively if rigorous evaluation is not carried out, since only evidence can drive and inform development. In our study, comparing the different scores over the period revealed a relevant change from the baseline to the last visit. This change was greatly due to counseling provided by community health workers during household visits, which proved to be impactful in creating and supporting sustainable lifestyle improvement.

The following findings can have practical implications, highlighting some key orientations and aspects for policymakers, governments, and funders:

1 Getting research into households: Despite the hype within the evidence revolution trends for getting research evidence into policy and practice, little attention has been paid to getting research evidence to consumers, who paradoxically are the final unit of evidence implementation. It is important for policymakers and researchers to consider directing more attention to innovative approaches for getting research into households/citizens.
2 The identification of PWD: In LMICs, in particular, identifying PWDs is still an issue. Most PWDs do not have any forms of identification. This issue precludes PWDs from benefiting from evidence-based policies aimed at improving their quality of life and increasing the equity gap. Personal identification of PWDs will allow them to gain access to basic services, participate in governance, and create bank accounts for personal expenses and/or businesses, which will all lead to them having a good life. This fact is aptly put in the words of a study participant:

*“Providing me with a disability card has enabled me to have my own identity, now I can have a bank account, access free services made available by the government, and live a good life.”*

*Young female with a disability (Bamenda, Cameroon).*

*“Using evidence-based guidelines as recommended by this pilot study will increase the identification of PWDs and increase their access to basic services.”*

*Regional Chief of PWDs, Ministry of Social Affairs, (Bamenda, Cameroon).*

3 Improving livelihoods through innovation and best practices: Using and integrating innovative approaches and best practices into households will improve livelihoods for PWDs, thereby reducing the equity gap. These best practices can range from inheritance for PWDs, access to financial institutions (including the digital economy), and inclusive governance, especially at decentralized levels.
4 Inclusive data collection and use for decision-making: While this innovative approach pushes relevant evidence into households, it also ensures that data from households reach decision-makers in health, education, empowerment, livelihoods, and social affairs departments. These real-time citizen science data can enable the inclusion of people with disability (and other target groups) into the data for the decision-making process. This will be a game changer for inclusive research and development if the effectiveness of innovation can be evaluated within a bigger study.
5 Community health workers can play a vital role that has been largely unexploited. Their household visits could potentially improve access to basic services for children with disabilities beyond just health. In this study, we see them supporting education, empowerment, social wellbeing, and livelihoods. This integrated approach makes them suitable for supporting people with disabilities.
6 This approach marks an innovative step to decolonizing aid for people with disabilities, especially in humanitarian settings. It is a context-relevant approach designed by experts and researchers including people with disabilities in this setting and using indigenous approaches that take into consideration the barriers and facilitators of the context—including conflict, disease, and fragility.
7 Using implementation science approaches will add value to existing policy and practice strategies and engage the public (citizens, households, and communities) in implementing evidence-based approaches and making evidence-informed choices for rights, needs, and basic services for people with disabilities. This will also help bridge the gap between research evidence and people with disabilities while adding a piece of the puzzle for evidence implementation for policies, practices, and the public.

Provided with more funding and global testing of this intervention in other parts of Cameroon and Africa at large, this approach will provide a robust evaluation of the intervention. In line with White’s opinion that evidence is the best buy in development (23), this study provides evidence of the presumptive idea that children and persons living with disabilities in Bamenda face many endeavors and are financially limited. Based on the global agenda of “Leave No-One Behind: Tackling Inequalities of Persons with Disabilities in the 2030 Agenda for Sustainable Development,” empowering children and persons with disabilities will bring forth an inclusive development goal (12, 27). PWDs are more likely to experience adverse socioeconomic outcomes, such as less education, poorer health outcomes, lower levels of employment, and higher poverty rates. As COVID-19 continues to have wide-reaching impacts globally, it is important to note how PWDs are uniquely impacted by the pandemic, including health, education, and transport considerations (28). Meanwhile providing medical assistance, educating them, and showing them love are protective factors to improve their wellbeing and livelihood. Unfortunately, most of these needs are neither being met by the government nor by the community. It is very important to pay attention to these persons for an inclusive development dominated by better standards of living (28). Knowledge brokering is very important for persons with disabilities in order to make informed choices and lend their voices to action plans and development programs without leaving anyone behind. Without voices and inclusive programs for persons with disabilities for inclusive development through innovation and best practices, development agencies and evidence to policymakers translators will fall short of bridging the gap of policy to practice gap. The statistical results from our intervention indicated a statistically significant impact of the intervention, which became stable after 4 months and was preserved until 12 months.

## Study limitations

Due to the sampling technique used to identify and sample PWDs, participants selected for this study may not be representative of all age groups and residential areas of children and adults living with disabilities in Bamenda; hence, our findings and the impact of this study cannot be generalized. Nevertheless, these findings can be still informative, providing current trends on the wellbeing of PWDs in Bamenda (Cameroon). In addition, due to limited funding, a greater reach of participants’ demands for the intervention was not attained for a high statistical power and a higher number of livelihoods affected. The available funding could support only 30 PWDs participating in the study out of the 56 children and persons living with disabilities recruited at the beginning of the study. Notwithstanding, the results obtained will be of great help in updating and aligning what needs to be done in future to further improve the livelihoods of children and adults living with disabilities. Provided more funding is allocated for this project, a more in-depth study with a higher sample size can be attained and more livelihoods of children and persons living with disabilities can be impacted in Bamenda and the whole of the North-West Region of Cameroon for an inclusive development based on the high demand of the intervention.

## Conclusion

PWDs still face challenges that hinder their full participation in society, which is further heightened by discriminatory attitudes and ableism. This culminates in marginalization and significant barriers to their inclusion. The extent of inequalities experienced by PWDs in all areas of development is often the result of shortcomings in attaining objectives in the areas in which they reside. The specific objectives of this intervention were to improve the livelihood of persons living with disabilities through household visits by community health workers to alleviate poverty and enable PWDs to live independently and make informed choices. Disability is a multi-dimensional and human rights issue. UNDP is working to support disability inclusive development and advance the rights of PWDs across the globe both in terms of mainstreaming and through targeted interventions. Effective Basic Services Africa is also in line with these visions and contributes to the attainment of the overall objective of the sustainable development goals. This intervention is recommended to be piloted in other parts of Cameroon and Africa in order to create a robust evaluation. One of the greatest challenges in rolling out this intervention to a greater sample size based on the demand for the intervention was limited funding. With the availability of more funding schemes, more livelihoods will be improved through economic empowerment activities, and research evidence will be documented for decision-making and inclusive development for all.

## Data availability statement

The original contributions presented in the study are included in the article/supplementary material, further inquiries can be directed to the corresponding author.

## Ethics statement

The study project protocol received full ethical clearance (approval code eBASE_IRB/02/2019). Informed, written consent was sought from study participants and/or their parents or legal guardians. The studies were conducted in accordance with the local legislation and institutional requirements. Written informed consent for participation in this study was provided by the participants’ legal guardians/next of kin.

## Author contributions

OP and MY developed the concept and protocol and performed data collection. OP supervised the operationalization of the project. NB, JK, ZM, OP, RM, GN, TK, and MY performed data analysis and developed the manuscript. All authors critically contributed by interpreting the results, commenting on the manuscript, and approving the final version of the manuscript.

## Footnotes

^1^ https://www.countrycoordinate.com/city-bamenda-cameroon/
